# Initial clinical applications treating pediatric and adolescent patients using MR-guided radiotherapy

**DOI:** 10.3389/fonc.2022.962926

**Published:** 2022-11-07

**Authors:** Margaret M. Kozak, David Crompton, Brandie A. Gross, Lyndsay Harshman, David Dickens, Jeffrey Snyder, Andrew Shepard, Joël St-Aubin, David Dunkerley, Daniel Hyer, John M. Buatti

**Affiliations:** ^1^ Department of Radiation Oncology, University of Iowa Hospitals and Clinics, Iowa, IA, United States; ^2^ Department of Pediatrics, the University of Iowa Hospitals and Clinics, Iowa, IA, United States; ^3^ Department of Hematology/Oncology, University of Iowa Hospitals and Clinics, Iowa, IA, United States

**Keywords:** MR guidance, pediatric, adolescent, cancer, radiotherapy

## Abstract

**Purpose:**

To demonstrate the clinical applications and feasibility of online adaptive magnetic resonance image guided radiotherapy (MRgRT) in the pediatric, adolescent and young adult (AYA) population.

**Methods:**

This is a retrospective case series of patients enrolled onto a prospective study. All pediatric (age < 18) and AYA patients (age< 30), treated on the Elekta Unity MR linear accelerator (MRL) from 2019 to 2021 were enrolled onto a prospective registry. Rationale for MRgRT included improved visualization of and alignment to the primary tumor, re-irradiation in a critical area, ability to use smaller margins, and need for daily adaptive replanning to minimize dose to adjacent critical structures. Step-and-shoot intensity-modulated radiation treatment (IMRT) plans were generated for all Unity patients with a dose grid of 3 mm and a statistical uncertainty of < 1% per plan.

**Results:**

A total of 15 pediatric and AYA patients have been treated with median age of 13 years (range: 6 mos - 27 yrs). Seven patients were <10 yo. The clinical applications of MRgRT included Wilms tumor with unresectable IVC thrombus (n=1), Ewing sarcoma (primary and metastatic, n=3), recurrent diffuse intrinsic pontine glioma (DIPG, n=2), nasopharyngeal carcinoma (n=1), clival chordoma (n=1), primitive neuroectodermal tumor of the pancreas (n=1), recurrent gluteo-sacral germ cell tumor (n=1), C-spine ependymoma (n=1), and posterior fossa ependymoma (n=1). Two children required general anesthesia. One AYA patient could not complete the MRgRT course due to tumor-related pain exacerbated by longer treatment times. Two AYA patients experienced anxiety related to treatment on the MRL, one of which required daily Ativan. No patient experienced treatment interruptions or unexpected toxicity.

**Conclusion:**

MRgRT was well-tolerated by pediatric and AYA patients. There was no increased use of anesthesia outside of our usual practice. Dosimetric advantages were seen for patients with tumors in critical locations such as adjacent to or involving optic structures, stomach, kidney, bowel, and heart.

## Introduction

There are approximately 12,000 children under the age of 18 diagnosed with cancer each year, of which about 3,000 will require radiotherapy (RT) (1). In adolescents and young adults (AYAs), defined as individuals between the ages of 15 and 39, there were approximately 80,500 new cancer cases diagnosed in 2020 (2). Advances in cancer care now yield excellent survival rates in pediatric malignancies (≥ 80%) (3). As a result, pediatric and AYA populations are at especially high risk of developing significant late effects of cancer treatment as compared to adult patients. It is therefore important to apply novel treatment modalities that simultaneously allow for tumor targeting and sparing of adjacent critical structures to optimize local control, prevent toxicity, and minimize risk for secondary malignancy in this vulnerable patient population.

Magnetic Resonance linear accelerator guided radiotherapy (MRgRT) enables high precision radiation delivery in a variety of clinical settings. To date, MRgRT approaches have been best described in adult populations, with most reports focusing on patients with abdominal and pelvic malignancies (4–6). MRgRT facilitates minimizing radiation dose to organs at risk (OARs) by allowing for daily online adaptive replanning. This enables the radiation oncologist to account for daily variations in patient and tumor anatomy, as well as smaller planning target volume (PTV) expansions due to the ability to account for these inter-fraction anatomical changes (7–9). Early data suggest a benefit in utilizing MRgRT for dose-escalation without a concomitant increase in treatment-related toxicity (10).

Available data on the use of MRgRT in the pediatric population is limited to a single case report of a 3-year-old girl with rhabdomyosarcoma of the diaphragm (11). This patient was treated with a smaller PTV volume than would have been acceptable with standard cone beam CT-based image guidance, thus sparing an additional 133 cm^3^ of normal tissue treated to the prescription dose than would have been possible in the absence of MRgRT.

Due to the limited data published on the topic of MRgRT in the pediatric population, as well as the unique advantages this approach affords, we compiled our institutional experience of treating young patients with this modality. Herein, we provide a retrospective case series of pediatric and young adult patients treated at our institution using MRgRT. In this review, we provide examples of cases that may benefit from MRgRT and describe the feasibility of treating pediatric and adolescent patients with this novel technology.

## Methods and materials

### Patients

We reviewed all pediatric and AYA patients at our institution treated on the Elekta Unity (Stockholm, Sweden) magnetic resonance image-guided linear accelerator (MR-Linac) since installation of the device. These patients were enrolled onto a prospective registry approved by our Institutional Review Board (IRB # 201109821).

### MR-guided radiotherapy system

The Elekta Unity MRgRT system consists of an integrated 7 MV flattening filter free linear accelerator and a 1.5 T Philips Magnetic Resonance Imager (Amsterdam, Netherlands). The Unity was originally described by Raaymakers et al. (12) and was commissioned for clinical use at our hospital in 2019 (13). The linear accelerator is positioned outside of the cryostat with a source axis distance of 143.5 cm and utilizes a 160-leaf multi leaf collimator, with the leaf travel in the superior/inferior direction, to define a maximum field size is 57.4 x 22 cm^2^. Volumetric Magnetic Resonance Images (MRI) were acquired of the patient directly before treatment and used to guide the development of adapted treatment plans. During treatment, cine images in three orthogonal axes (axial, coronal, sagittal) were acquired with a frame rate of 5 frames/second.

The Unity system is used in combination with the Elekta Monaco treatment planning system (v5.40.01) which employs a Monte Carlo dose calculation algorithm to account for the effects of the magnetic field on the dose distribution. Step-and-Shoot intensity-modulated radiation treatment (IMRT) plans were generated for all Unity patients with a dose grid of 3 mm and a statistical uncertainty of 1% per plan.

### Simulation and treatment planning

All patients underwent treatment planning computed tomography (CT) and Magnetic Resonance imaging in accordance with our standard institutional protocol. Positron Emission Tomography (PET)/CT simulation or diagnostic images were fused for treatment planning for some of the cases. For each patient, CT and MRI were acquired in the treatment position using an immobilization device appropriate for the area of treatment. Target volumes and OARs were contoured on the CT, MRI and PET images and used to create structures (OARs and GTVs) on the CT. A coplanar beam arrangement of 11-19 beams was used. The beams were evenly distributed with special consideration given to avoid the cryostat pipe of the Magnetic Resonance Imager and the high-density regions in the couch. For Case 1, a comparator plan was generated in Pinnacle (Version 16.2) using inverse planning with two full arc VMAT beams. An adaptive convolve algorithm was used for dose calculations in the VMAT plan.

At the first daily treatment, the patient was positioned using the immobilization equipment used during the simulation process and an MRI was obtained. A rigid registration was performed between the original CT and the daily MRI, to evaluate the agreement between the reference plan contours and the daily changes in internal anatomy. If no substantial changes are noted, an “adapt-to-position” treatment plan was generated, the goal of which is to reproduce the dose distribution of the reference plan while accounting for the translational shifts of the patient. In this workflow, the calculation is performed based on the density map of the original simulation CT that is rigidly registered to the daily MRI.

If substantial changes between the reference plan contours and the daily anatomy were noted, an “adapt-to-shape” treatment plan was generated. Contour adaptation was aided through deformable image registration to propagate contours from the reference plan to the daily MRI. A bulk density assignment of all delineated structures was done to allow dose calculation to be performed directly on the daily MRI. The density assignments were defined during the initial treatment planning process and were propagated from the simulation CT or set manually. Physicians were required to verify the registration and the adapted contours. If necessary, recontouring (editing) of the target volume and/or OARs was performed by the physician at the treatment console.

In either workflow, three patient specific quality assurance (QA) steps were performed prior to initiating an adaptive treatment. The first QA step is visual inspection of the electron density map to verify that the electron density assignments were within expected values. The second step was a secondary calculation of a point dose in the target volume using Radcalc software v6.3 (Lifeline Software Inc., Tyler, TX) (14), which was calculated per beam and for the total plan. At our institution, we require the composite Radcalc dose to be within 5% of the treatment plan dose for the plan to pass the second check. The third step was a data transfer integrity check, also using Radcalc, to verify that the treatment parameters in Mosaiq match the RT plan DICOM file.

## Results

Our first patient was treated with MRgRT in May 2019. To date, we have treated 15 pediatric and AYA patients (**Table 1**). The median patient age was 13 years (range: 6 mos - 27 years). The clinical settings in which MRgRT was recommended included: Ewing sarcoma (primary and metastatic, n=3), recurrent diffuse intrinsic pontine glioma (DIPG, n=2), nasopharyngeal carcinoma (n=1), retroclival chordoma (n=1), primitive neuroectodermal carcinoma of the pancreas (n=1), recurrent gluteo-sacral germ cell tumor (n=1), Wilms tumor with unresectable inferior vena cava (IVC) thrombus (n=1), recurrent post-transplant related lymphoproliferative disease (1), spinal glioma (1), spinal cord ependymoma (n=1), and posterior fossa ependymoma (n=1). In these patients, a total of 388 fractions of radiation therapy were delivered. This includes 6 fractions as part of SBRT plans and 382 conventionally fractionated regimens.

**Table 1:** Characteristics of patients treated with MR guided radiotherapy.

Age, sex	Diagnosis	Radiotherapy dose	Rationale for MR-Linac use	On-treatment modifications
6 mos, M	Orbital chloroma, relapsed AML	4 Gy in 2 fractions	Superior visualization of and alignment to tumor, smaller PTV margins	None
2, F	Wilms tumor, subtotally resected with extensive IVC tumor thrombus	19.8 Gy in 11 fractions	Tumor thrombus not well visualized on conventional CT imaging; treatment on MR-Linac allowed for smaller margins and improved sup-inf visualization of thrombus for daily patient positioning	None
7, M	Primary spinal cord glioma involving T1-T5	50.4 Gy in 28 fractions	Improvement in ability to visualize and align to the primary tumor each day	None
8, M	T4N2M0 nasopharyngeal carcinoma	66 Gy in 33 fractions	Allowed for improved visualization of involved LNs and evaluation of their change in size over the course of treatment as well as their relationship to the primary tumor; daily cord visualization; superior alignment to base of skull as compared to standard cone beam CT	Incorrect flexion of the neck or shoulder rotation was noted on two separate occasions; patient was repositioned and re-imaged prior to starting treatment
8, M	Yolk sac tumor of sacrococcygeal region	54 Gy in 28 fractions	Primary tumor not well seen on CT images; MRI allowed for superior daily positioning especially with respect to the rectum	None
9, M	Retroclival chordoma	60 Gy in 50 BID fractions followed by 16.8 Gy in 14 BID fractions boost	Daily alignment to primary tumor rather than clivus was superior with MRI as compared to daily cone beam CT; allowed for improved sparing of the brainstem	On one occasion, patient was noted to have moved during the adaptive replanning process; a repeat cone beam was obtained, and treatment delivered appropriately
13, M	Recurrent diffuse intrinsic pontine glioma	Re-irradiation to 31.2 Gy in 26 BID fractions	Re-irradiation in critical area with improved visualization of brainstem for daily image guidance; smaller PTV margins	Patient complained of mask being too tight; had to make adjustments to the mask and repeat MR twice for proper positioning on the first day of treatment
16, M	DLBCL of the jejunum	45 Gy in 25 fractions	Superior visualization of treatment area; adaptive replanning used to minimize dose to the stomach and contralateral transplanted kidney	Patient was found to have moved during the adaptive replanning process; a repeat MR was obtained and plan re-optimized and delivered appropriately
19, M	Recurrent diffuse intrinsic pontine glioma	Re-irradiation to 31.2 Gy in 26 BID fractions	Re-irradiation in critical area with improved visualization of brainstem for daily image guidance; smaller PTV margins	Head rotation noted on daily setup imaging on two separate occasions requiring patient adjustment and re-imaging prior to treatment
19, F	Ewing sarcoma of the sacrum	55.8 Gy in 31 fractions	Superior visualization of primary tumor for daily alignment as compared to cone beam CT	None
23, M	Posterior fossa ependymoma	59.4 Gy in 33 fractions	Superior visualization of the brainstem for daily alignment with goal to keep hot spots off of critical structures	None
24, M	Metastatic Ewing sarcoma involving gluteal muscle	SBRT 24 Gy in 3 fractions	Tumor not seen on standard CT imaging; due to location in gluteal musculature required adaptive replanning for each fraction	None
24, M	PNET of pancreas	48.6 Gy in 27 fractions	Superior visualization of primary tumor; improved sparing of adjacent duodenum	None
25, M	C-spine ependymoma	50.4 Gy in 28 fractions	Superior visualization of the spinal cord; goal to keep hot spots off of the cord	Patient was found to have moved during the adaptive replanning process, a repeat MR was obtained and no significant changes were made to the plan
27, F	Ewing sarcoma of C1 vertebral body	50.4 Gy in 28 fractions (original prescription to 55.8 Gy in 31 fractions)	Superior visualization of primary tumor and relationship to nearby critical head and neck/base of skull structures as well as the spinal cord	Patient had significant neck pain and had to get off the treatment table on two separate occasions; ultimately could not complete treatment on the MR-Linac due to length of treatment delivery and was transitioned to a conventional linear accelerator

Initial concerns included the potential inability of younger children to tolerate the longer times required for MRgRT treatment delivery. For patients treated on the Unity, the average treatment time was 45 minutes per fraction for adapt-to-shape, and in this cohort of patients, 172 total fractions were delivered with this workflow. We found that average treatment time for the adapt-to-position workflow was 36 minutes per fraction and this accounted for a total of 216 fractions in this cohort of patients. Ultimately, 169 successful fractions of treatment in 7 patients under 10 years old was well tolerated.

In contrast to what might be expected, we found that our AYA patients were more likely to have difficulty with treatment than our pediatric patients. One young adult female patient had to be transitioned to a conventional linear accelerator due to tumor-related pain exacerbated by longer treatment times. Two young adult male patients experienced anxiety related to claustrophobia – one received a stress ball to squeeze during his time on the treatment table and the other required daily Ativan for the entirety of his treatment course. No pediatric patients experienced any difficulties with the longer treatment times that are characteristic of MRgRT delivery, and anesthesia was only required for one very young child aged 14 months.

Two children required general anesthesia for the duration of their radiation treatment. Our current treatment vault is set up with piped gasses into the room and MR-compatible anesthesia equipment available in the vault. We did not experience any problems with which would compromise the anesthesia delivery for the two young patients who required this as part of their treatment.

Perceived advantages to MRgRT ranged from superior visualization of tumor due to enhanced soft tissue imaging allowing for improved patient positioning, daily online treatment adaptation with avoidance of nearby OARs, and daily adapt-to-shape for tumors that changed in size and position or for OARs that moved into the high dose treatment field. Four representative cases will be discussed in detail below.

### Improvement in soft tissue imaging and patient positioning

A 14-month-old girl presented with progressively worsening abdominal distention, decreased oral intake, and listlessness. CT imaging showed a large, heterogenous retroperitoneal mass without identifiable normal left kidney. There was renal parenchymal opacification, an extensive IVC thrombus extending from the hepatic confluence to the right iliac vein and pulmonary and liver metastases. Urine catecholamines were not elevated. Together, these findings suggested Wilms tumor. She was started on neoadjuvant chemotherapy after which she underwent successful resection of the primary mass. The residual tumor thrombus was unresectable and remained in the IVC. Pathology showed favorable histology Wilms tumor.

Adjuvant radiotherapy to the flank and residual tumor thrombus was recommended to a dose of 10.8 Gy in 6 fractions of 1.8 Gy to be followed by a 10.8 Gy in 6 fractions boost to the unresectable IVC tumor thrombus for a total dose of 21.6 Gy in 12 fractions. A 4-dimensional CT was obtained for radiation treatment planning. She received her initial flank treatment on a conventional linear accelerator. MRgRT was selected for boost treatment due to superior visualization of, and alignment to the IVC tumor thrombus in the superior-inferior direction and for intrafraction motion management. Tumor extent in the IVC was poorly appreciated on CT imaging, however, thrombus was well visualized on T2 MRI. This enabled the use of smaller treatment margins. In addition, treatment on the MR-Linac also allowed for better evaluation of dose to the contralateral kidney – information that would be lost if treating on a conventional linear accelerator. Daily anesthesia was required for each treatment due to the young age of the patient.

**Figure 1:**
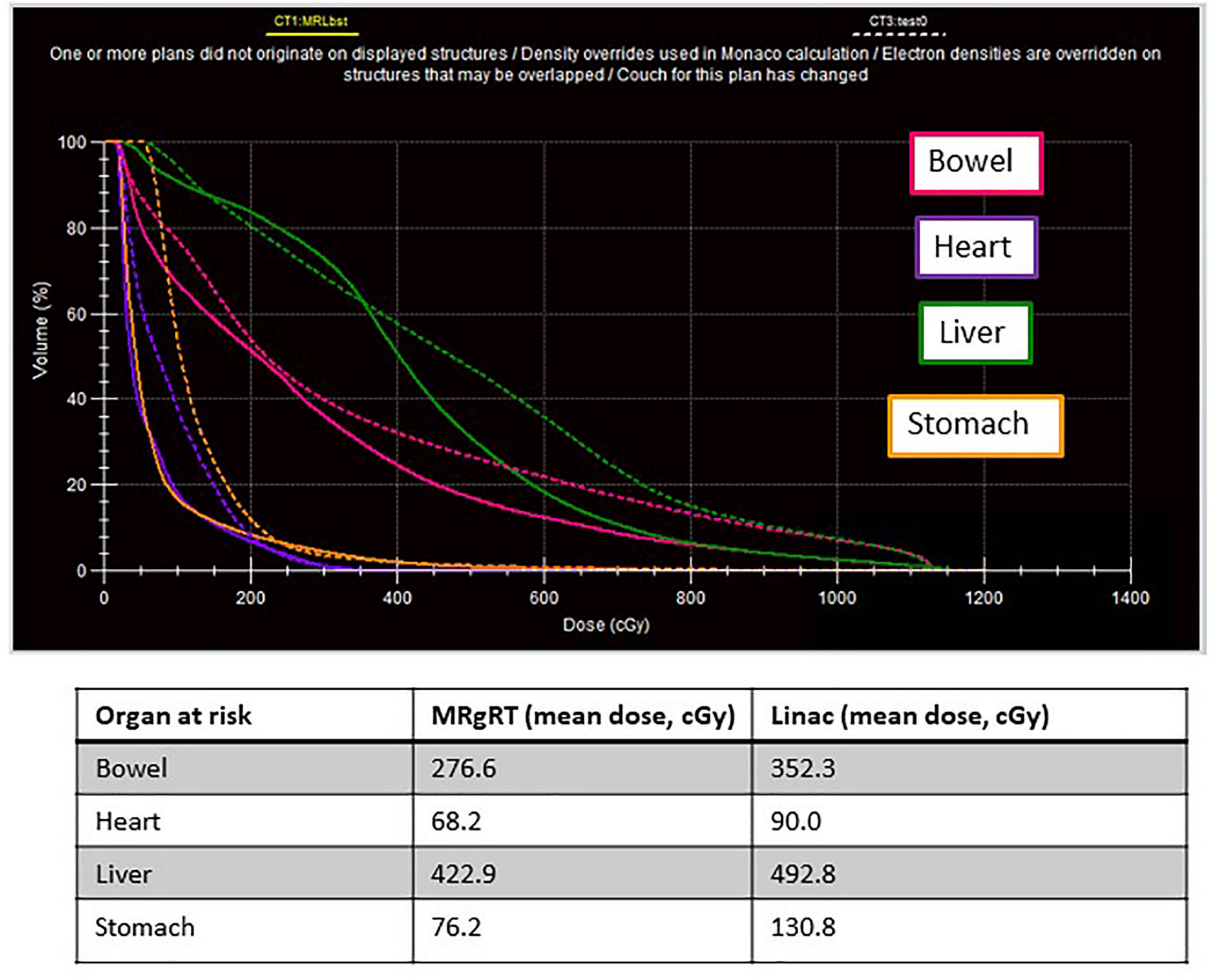
A 14-month-old female with favorable histology Wilms tumor and an unresectable IVC tumor thrombus underwent adjuvant flank radiation to 10.8 Gy followed by a boost to residual disease to 19.8 Gy. This figure represents the dose volume histogram comparing the MRL plan (solid line) with the conventional linac plan (dashed line). Organs at risk are labeled with corresponding colors.

An alternative plan for treatment delivery on a conventional linac was generated for comparison with the MRgRT plan. The boost volume was defined as IVC thrombus plus a 3 mm PTV for the MRgRT plan versus a 5 mm CTV margin and 5 mm PTV margin as per standard Wilms protocols. A dose volume histogram depicting the comparison of the MRgRT plan with smaller margins and the conventional linear accelerator plan is depicted. Mean doses to adjacent critical structures including the bowel, heart, liver and stomach were all decreased with MRgRT (**Figure 1**).

The patient tolerated the treatment well under general anesthesia. Daily adaptive replanning was performed over the course of her treatment but no adapt-to-shape was needed. The patient has stable disease now 15 months after completing all therapy. Her case has been discussed on the national level and it is felt that watchful waiting is the most appropriate course of action at this time. No significant toxicities have been experienced to date in early follow-up.

### Online treatment adaptation based on changes in organ at risk anatomy

A 16-year-old male with a history of end stage kidney disease secondary to posterior urethral valves for which he underwent kidney transplant in 2005 presented with two-month history of fatigue, epigastric pain, and black tarry stools. He was found to be anemic and underwent upper endoscopy and colonoscopy which revealed a gastric ulcer for which he was started on high dose of proton pump inhibitor. His symptoms progressed to significant abdominal pain. A capsule endoscopy showed an obstructive mass in the jejunum precluding passage of the pill. He underwent laparoscopy and mesenteric mass biopsy with flow cytometry consistent with post-transplant lymphoproliferative disorder. Lumbar puncture and bone marrow biopsy were negative. A PET scan showed two hypermetabolic masses within the jejunum, the larger of which was causing upstream obstruction and dilation. Due to significant and poorly controlled abdominal pain as well as trapped pill endoscopy, small bowel resection was performed. Final pathology showed a monomorphic B-cell post-transplant lymphoproliferative disorder consistent with diffuse large B cell lymphoma (DLBCL), germinal center type.

He was started on cyclophosphamide, vincristine, Adriamycin, prednisone, cytarabine, asparagine and intrathecal methotrexate chemotherapy as per CCG5961 (15). A post-treatment PET scan did not show evidence of disease recurrence, however, 14 months later he presented again with declining hemoglobin and an episode of tarry stools. Ultimately, imaging revealed locoregional tumor recurrence. He received 4 cycles of weekly rituximab followed by radiation treatment to a dose of 45 Gy in 25 fractions to two recurrent jejunal masses.

Motion of the target volume was evaluated using 4-dimensional CT at the time of initial radiation treatment planning. A treatment planning MRI was also obtained and the jejunal masses were best seen on T1-pre-contrast images. The masses did not move significantly with breathing and therefore a motion-inclusive internal target volume (ITV) was created, followed by a 5 mm PTV expansion. The dose constraint to the stomach was set as a maximum density (Dmax) of 39 Gy, the jejunum was constrained to Dmax of 48 Gy, and the transplanted kidney, located in the contralateral pelvis, was kept to a Dmax of 2 Gy. A total of 11 beams were used to construct an IMRT plan using the Monaco treatment planning software.

**Figure 2:**
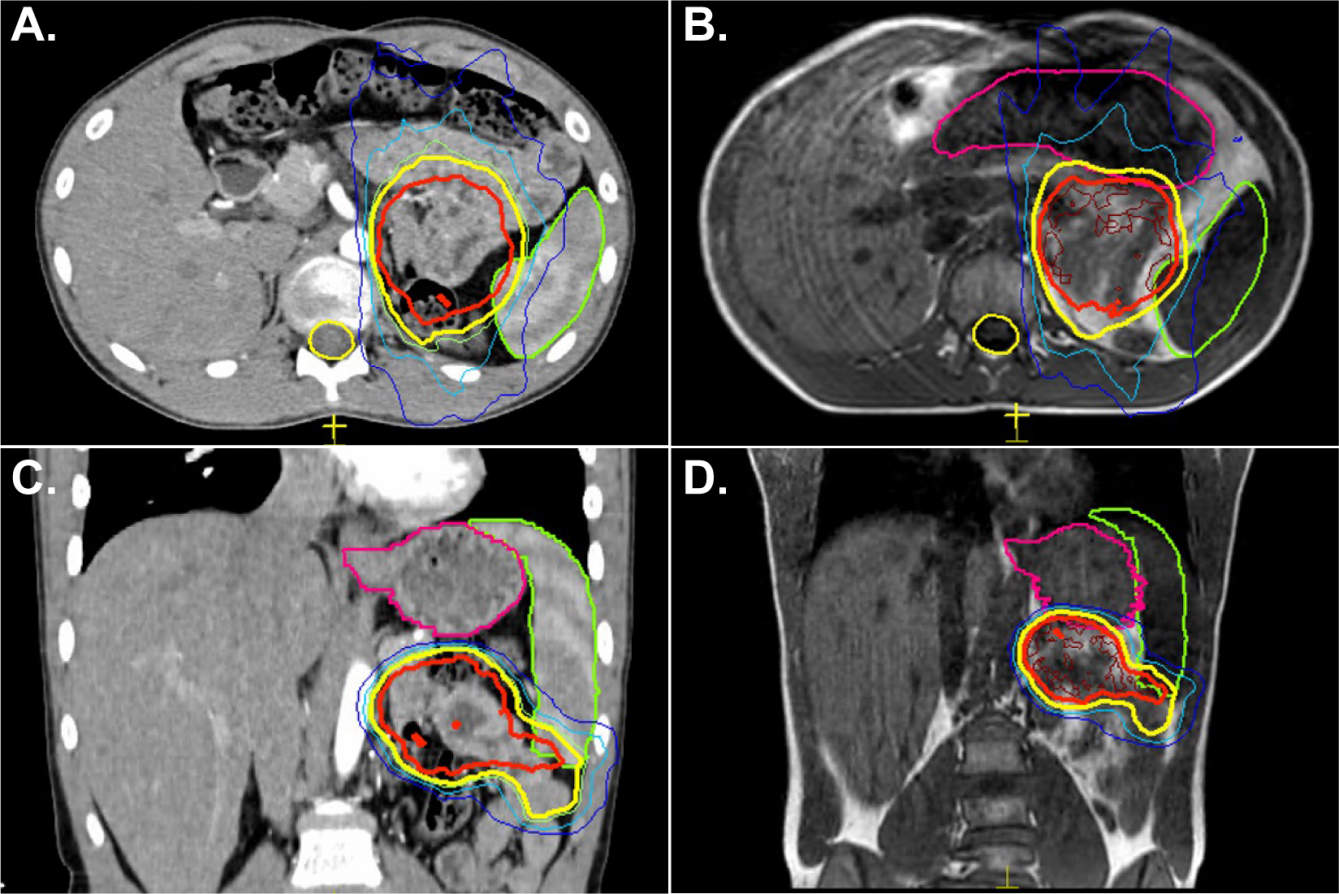
A 16-year-old male with history of kidney transplant and lymphoma presented with tarry stools and was found to have lymphoma recurrence in the jejunum as the only sites of disease. He received chemotherapy followed by radiation treatment to 45 Gy in 25 fractions. Panel **(A)** shows the radiation treatment plan on CT where the structure in green represents the spleen, the yellow line represents the 39 Gy isodose line, and the red line represents the 45 Gy isodose line. Panel **(C)** shows the initial treatment plan and the relationship to the stomach, outlined in pink. Panels **(B, D)** are a representative example of change in stomach shape while the patient underwent treatment. Without adapt-to-shape capability, the stomach would have received a higher dose than desired.

Due to changes in the shape of the stomach, adapt-to-shape was performed several times over the course of treatment – this allowed for keeping the PTV at 5 mm rather than using a larger PTV which would have been required on a conventional linear accelerator. **Figure 2** shows the initial treatment plan and the right panel shows how change in stomach shape would have prevented meeting dose constraints had adapt-to-shape not been employed. In one instance, the patient was found to have moved during the adaptive re-planning process. A repeat MR was obtained, and the plan re-optimized a second time and delivered appropriately. He is now doing well nearly one year since completing therapy. He was able to resume competitive high school athletics within a month of completing therapy. No sequelae have been reported to date and he continues to have excellent kidney transplant function.

### Adaptive replanning for daily gross target volume shape change

A 24-year-old male with history of metastatic Ewing sarcoma presented with painful bilateral gluteal metastases. He had undergone chemotherapy and surgical resection of his primary gluteal mass with negative margins and 40% tumor necrosis 1 year prior. He developed metastatic progression with osseous metastases in the left distal femur and right humerus as well as a right paraglutaeal subcutaneous and left intragluteal metastases. After 3 cycles of topotecan and cyclophosphamide, his bony disease resolved but the gluteal metastases demonstrated interval growth. He was recommended to undergo palliative radiotherapy to a dose of 24 Gy in 3 fractions, with plans to be treated with MRgRT for superior visualization of the target volumes.

**Figure 3:**
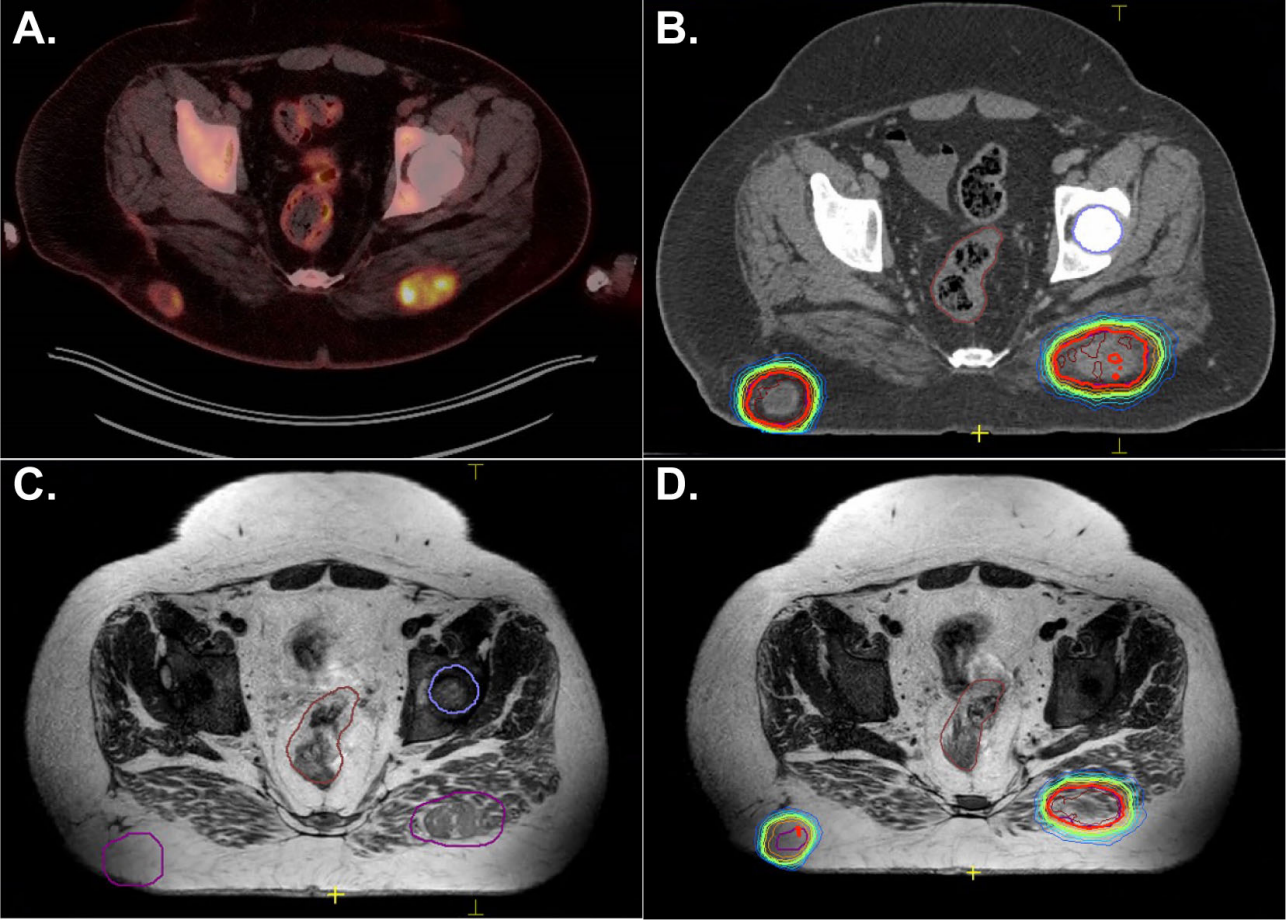
A 24-year-old male with metastatic Ewing sarcoma presented with two enlarging, painful gluteal metastases. Panel **(A)** shows the diagnostic PET scan. Panel **(B)** shows the lesions on CT. Notably, on both the PET scan and treatment planning scan, both nodules were visualized in the same plane. A single isocenter plan was created. Panel **(C)** shows the MRI obtained during the first fraction of treatment where the right-sided lesion is now out of the plane. Panel **(D)** shows successful adapt-to-shape for this single isocenter plan.

A single isocenter plan was created treating both lesions simultaneously each day. Adapt-to-shape was required for all 3 fractions. **Figure 3** shows the initial pre-treatment PET scan and treatment planning CT. The left lower panel shows an acquired MR image where the right-sided lesion is no longer in the same plane as the left-sided lesion. Adapt-to-shape allowed for the creation of a new plan in real time with adequate coverage of both sites of disease. These lesions were poorly visible on non-contrasted CT and hence improved targeting was realized with the use of MRgRT. The patient had good initial local response to disease without sequelae but has succumbed to his metastatic disease.

### Daily adapt-to-shape to reduce high dose to critical structures

**Figure 4:**
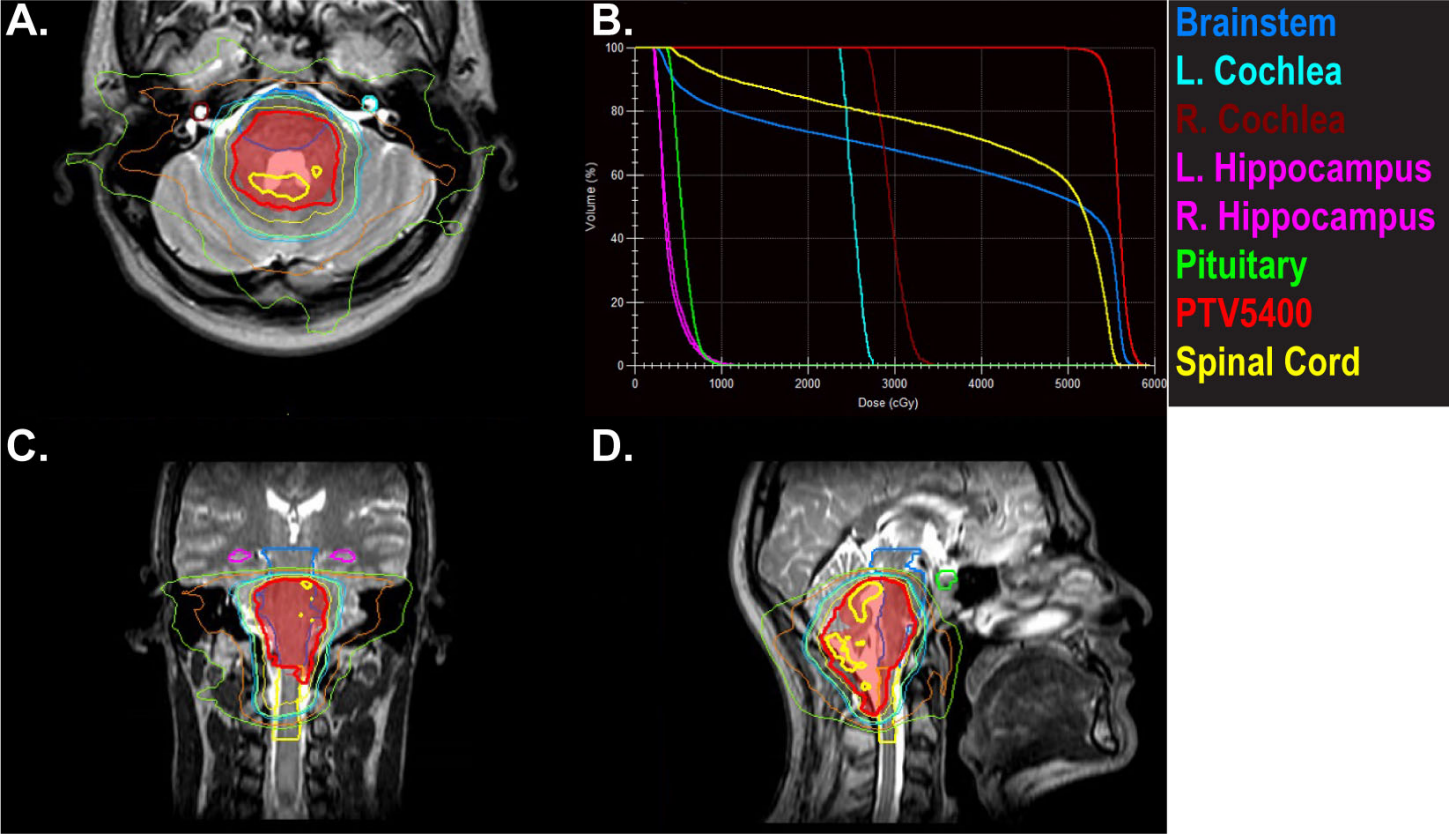
A 22-year-old male with posterior fossa ependymoma underwent near total resection and adjuvant radiotherapy to 54 Gy followed by a cone down boost to 59.4 Gy. Panels **(A, C, D)** depict the initial plan to 54 Gy. The hot spots of 57 Gy (thick yellow line) are kept off the brainstem andspinal cord. The PTV is in red color wash. Panel **(B)** shows doses to organs at risk that were prioritized during the treatment planning process and during daily adapt-to-shape MRgRT.

A 23-year old male patient presented with a month-long history of progressively worsening headaches and nausea that progressed to early morning vomiting. He was treated with conservative measures without improvement. An MRI brain revealed a posterior fossa mass originating in the floor of the fourth ventricle. He was taken to the operating room for tumor resection, with unresectable disease left behind involving the brainstem best seen on T2 images. Pathology revealed grade 2 ependymoma without anaplastic features, PFB subtype. He was simulated with an MR-Linac-compatible face mask. The postoperative tumor bed and residual disease was contoured and defined as the CTV. The PTV was a 3 mm expansion on the CTV. Postoperative radiotherapy was planned to a dose of 54 Gy followed by a cone down boost to 59.4 Gy with exclusion of the upper cervical cord. Goals were to keep the brainstem to less than 107% of the prescription dose and the upper cervical cord to <56 Gy. An 11 field IMRT plan was created (**Figure 4**).

A T2 MRI was obtained for each treatment and alignment was performed to the brainstem and spinal cord. Daily adapt-to-shape was required to keep the 57 Gy line off the brainstem and spinal cord. Verification images were taken to ensure that the patient had not moved in position during the adaptive re-planning process and cine MRI confirmed lack of movement during the treatment. The patient experienced anxiety related to wearing the face mask and required daily Ativan at the beginning of his treatment. His anxiety improved as he progressed through the course of radiation treatment and did not necessitate transitioning to a standard linear accelerator. The patient is currently 1 month since completing therapy with no evidence of recurrence and no significant sequelae.

## Discussion

Pediatric and young adult radiotherapy presents unique challenges to the practicing radiation oncologist. Improvements in outcomes of pediatric cancers over the past several decades, with survival rates **>** 80%, means that patients have an increased risk of late effects and secondary malignancy. Late effects from radiotherapy relate to the location of the primary tumor being treated, the total radiation dose, as well as the age of the patient, and are therefore variable. They can include anything from neurocognitive effects of brain radiation, to cardiac dysfunction, small.bowel obstruction, and bony growth abnormalities. MRgRT affords a unique opportunity for superior visualization of the tumor and enables the treating radiation oncologist to optimize treatment plans in real time. In addition, it allows for smaller PTV margins and the ability to better align to tumors that may change in shape or position over the course of the treatment. For centers without access to protons or for patients unable to travel, MRgRT could benefit the practice and patient by allowing for individualized daily treatment, minimization of PTV margins, and decreasing dose to critical structures.

To the best of our knowledge, we present the largest series of pediatric and AYA patients treated using MRgRT in the United States. Perhaps one of the most important findings of our study is that treating pediatric and AYA patients on the MR-Linac is feasible, was well tolerated overall, with all but one patient completing their treatment course without interruptions. Based on our experience, we recommend that radiation oncologists consider treating pediatric and AYA patients using MRgRT if the clinical scenario permits.

Not all pediatric and AYA patients will be candidates for MRgRT. Factors such as longer time under anesthesia, large field sizes, and complexity of treatment volumes not allowing for easy online adaptive replanning can hinder the effective use of MRgRT in this patient population. For many pediatric radiation oncology scenarios that utilize relatively low doses and standard treatment fields, such as for Wilms tumor or whole lung radiation for patients with metastatic Ewing sarcoma, there is likely no significant advantage to MRgRT except in special circumstances. In this report we present such a unique case in which an unresectable tumor thrombus was better visualized on the MR-Linac and allowed for improved daily patient positioning and better avoidance of nearby organs at risk. However, for large field treatments such as whole lung, whole abdomen, some flank treatments, and many extremity sarcomas, MR-guidance is not feasible. Patients with bony metastases may benefit from MRgRT depending on the location being treated. For example, tumors abutting the orbit, or located in the clivus or sacrum can be considered for SBRT using MRgRT. In addition, spinal cord or spinal canal metastases may benefit from the superior visualization and alignment that MRgRT affords, especially if the clinician is seeking to dose escalate.

In conclusion, in this review we present the largest series of pediatric and AYA patients treated with MRgRT. Further study is warranted to assess which patients will obtain the most significant benefit from this approach. In addition, de-escalation of treatment by progressively shrinking gross tumor margins or dose de-escalation in the event of significant tumor response are other avenues to be explored in the pediatric MRgRT setting.

## Data availability statement

The original contributions presented in the study are included in the article/supplementary material. Further inquiries can be directed to the corresponding author.

## Ethics statement

Written informed consent was obtained from the individual(s), and minor(s)’ legal guardian/next of kin, for the publication of any potentially identifiable images or data included in this article.

## Author contributions

MK and AS - conception, data compilation, writing, editing. DC - data compilation, writing. BG - image design, writing. LH - data compilation, writing. DDi - data compilation, writing. JS - data compilation, writing. JS-A - data compilation, writing. DDu - data compilation, writing. DH- conception, data compilation, design, writing. JB - conception, data compilation, design, writing. All authors contributed to the article and approved the submitted version.

## Conflict of interest

MK has received honorarium from Elekta. DH is a consultant for Elekta.

The remaining authors declare that the research was conducted in the absence of any commercial or financial relationships that could be construed as a potential conflict of interest.

## Publisher’s note

All claims expressed in this article are solely those of the authors and do not necessarily represent those of their affiliated organizations, or those of the publisher, the editors and the reviewers. Any product that may be evaluated in this article, or claim that may be made by its manufacturer, is not guaranteed or endorsed by the publisher.
